# Cardiometabolic risk factors and social jetlag in university professors

**DOI:** 10.1590/1414-431X2023e12539

**Published:** 2023-06-30

**Authors:** D.M.L. Galeno, H.J.A. Peixoto, B.T.S. Carneiro, M.A. Leocadio-Miguel

**Affiliations:** 1 Departamento de Fisiologia e Comportamento, Universidade Federal do Rio Grande do Norte, Natal, RN, Brasil; 2 Department of Psychology, Northumbria University, Newcastle upon Tyne, United Kingdom

**Keywords:** Stress, Social jetlag, Sleep, Cardiometabolic disorders, University professors

## Abstract

Chronic stress leads to circadian disruption, with variability in sleep time and duration. This scenario increases the prevalence and incidence of cardiometabolic abnormalities. Social jetlag (SJL), a proxy of circadian disruption, has been associated with increased vulnerability to the development of metabolic syndrome, obesity, and type 2 diabetes. This research aimed to evaluate how variables associated with cardiometabolic risk are related to SJL and poor sleep among university professors. From 2018 to 2019, full-time university professors (n=103) with a mean age of 44±5.4 years were assessed for sleep quality, chronotype, SJL, metabolic components, sociodemographic characteristics, and physical evaluation. Sleep quality and weekday sleep duration were associated with stress (r=0.44 and r=-0.34) and anxiety (r=0.40), respectively. Mean sleep duration (n=65) was 7.0±1.1 h and all professors with poor sleep (41.2%; n=28) worked 40 h/week. Professors who slept less were significantly (r=-0.25) older, and teaching time (years) was positively correlated with blood glucose (r=0.42). Mean SJL was 59.8 ±4.5 min (n=68) and 48.5% of these professors had values ≤1 h and 51.4% ≥1 h. SJL and blood glucose concentration were associated (r=0.35), which reinforced that challenges to the circadian system reverberate on metabolism. In this study, professors at the Federal University of Rio Grande do Norte had cardiometabolic risks related to anxiety, stress, and sleep quality.

## Introduction

Professors are prone to occupational stress (1) and their work routine threatens the circadian system, causing adverse health outcomes (2). There is now increasing evidence of a link between disturbances in circadian rhythm and metabolic syndrome leading to cardiometabolic diseases and comorbidities (3).

Stress can be defined as any stimulus or experience that threatens homeostasis and refers not only to challenges imposed on an organism by the external or internal environment, but also describes the processes an organism employs to cope with different demands (4). Teaching can be considered a demanding and stressful profession that undeniably influences the duration and quality of sleep, affecting both physical and psychological health and impacting work performance (5).

Mental workload refers to the tensions that work demands cause in the worker and may be associated with the presence of multiple stressors related to constant demands for teaching improvement, academic competition, interpersonal relationship, and psychological loads (1).

Social demands (work, school, family) and incompatibility of endogenous rhythms may lead to circadian misalignment that can be related of several factors, such as short weekday sleep duration, poor sleep, altered eating patterns, sleep disorders, and an extreme evening chronotype (behavioral preference for a late bedtime (6). Incompatibility between endogenous rhythms and social jetlag (SJL) describes and quantifies the chronic misalignment between an individual's biological clock and social clock in 7 days. It's defined as the absolute difference between the mid-sleep phase on weekends and weekdays (7).

The American Academy of Sleep Medicine (AASM) recommends 7 or more hours of sleep per night to promote optimal health among adults aged 18 to 60 years (8). Insufficient sleep is of particular concern because experimental and epidemiological data have linked endocrine dysfunction to changes in sleep behavior, including chronic sleep restriction, daytime sleepiness, insufficient and/or excessive sleep duration, and changes in sleep architecture. This can lead to symptoms of stress and anxiety, two established risk factors for obesity, diabetes, and higher risk of incident cardiovascular disease (9) and all-cause mortality (10).

Insomnia or circadian disorder is associated with high sleep reactivity (degree to which stress disrupts sleep, resulting in difficulty falling asleep and staying asleep), risk of shift work disorder, cognitive and neuropsychological dysfunctions, even increasing risk of accidents, suicidal ideation and behaviors (11), and depression and anxiety (12). In addition to sleep duration, high variability in sleep timing is also associated with higher prevalence and incidence of metabolic abnormalities (13,14).

The higher the demand for work or study, the greater the variability in sleep timing (15). Widespread nighttime light and locomotor activity have significantly changed human sleeping patterns and increased the odds for circadian disruption, factors that induce changes in metabolism and energy homeostasis (16).

Although many studies have addressed the association between circadian disruption and increased risk for cardiometabolic diseases, the relationship among sleep duration, social jetlag (SJL), stress, and anxiety in university professors is not fully understood. We hypothesized that anxiety and stress symptoms in the work environment are associated with social jetlag, poor sleep, and greater cardiometabolic risk factors in university professors.

## Material and Methods

### Participants

This research was conducted from January 2018 to November 2019. The sample consisted of full-time professors selected through random stratification sampling, with probability proportional to the size of this study. The inclusion criteria of the sample were professors of both sexes, aged between 30 and 65 years, who were part of the permanent staff of professors at the Universidade Federal do Rio Grande do Norte (Brazil).

### Study design

This was a descriptive, cross-sectional study. Initially, we performed a physical evaluation to collect anthropometric data and biochemical profile. Professors were invited to respond to validated forms and scales online. The project was submitted and approved by the Research Ethics committee (2.401.132) of this university and conformed to international ethical standards based on the Declaration of Helsinki. All volunteers signed an informed consent to participate.

The evaluation of sleep quality was performed using the Pittsburgh Sleep Quality Index (PSQI) (17), which has seven components: 1) subjective quality of sleep; 2) sleep latency; 3) duration of sleep; 4) habitual sleep efficiency; 5) sleep disorders; 6) use of sleeping pills; and 7) daytime sleepiness and daytime disturbances. Each part has specific scores, with 21 points being the maximum score. Scores greater than five indicate poor sleep quality. Sleep latency is the time a person takes between turning off the lights and actually falling asleep. Sleep efficiency is the percentage of time spent asleep while in bed. A normal sleep efficiency is considered to be 85% or higher.

Chronotype classification and SJL analyses were performed using the Munich Chronotype Questionnaire (MCTQ). The mid-sleep phase corrected (MSFsc) was used as a chronotype measurement and calculated by the formula MSFsc = MSF - 0.5*[SDf - (5*SDw + 2*SDf) / 7] where MSF is mid-sleep on free days, SDf is sleep duration on free days, and SDw is sleep duration on working days (7).

SJL was calculated as the absolute difference between sleep midpoint on weekends and weekdays (MSF−MSW). Sleep duration was obtained by the mean sleep duration in working days and free days, assuming five working days and two free days per week as standard (14). We also obtained information about anxiety symptoms and perceived stress through validated questionnaires (18,19). Sociodemographic data, presence of comorbidities, commuting to and from work, degree of psychological tension, sleep latency, use of sleep aid medication, naps (brief sleeps), alcohol consumption, self-reported nocturnal intake of calories, arterial blood pressure, blood glucose concentration, high-density lipoprotein (HDL), low-density lipoprotein (LDL), triglycerides, body mass index (BMI), waist circumference (WC), and factors related to teaching, work, and daily life were collected.

### Statistical analyses

Data analyses were conducted according to the probability distribution of data. We used Pearson's correlation and Student's *t*-test for normal distribution and Spearman's correlation for non-normal distribution. Data are shown as scatter plots (correlations) or as means±SD (normal distribution). Correlations and differences between groups were considered statistically significant when P<0.05.

## Results

A total of 103 professors answered the online questionnaires, of which only a part attended the physical assessment session and delivered the complementary exams. Most of the professors (n=65) worked 40 h/week. Of these, 39.1% were adjunct professors (n=27), 44.9% associate professors (n=31), and 8.6% full professors (n=6). The main self-reported comorbidities in the present study were anxiety (16.1%), depression (10.2%), hypercholesterolemia (16.1%), and hypertension (23.5%). The general and teaching characteristics of professors are described in Table 1.

**Table 1:** Sociodemographic data and teaching characteristics of professors.

Characteristics	n (percentage)
Gender	n=103
Male	41 (39.81 %)
Female	62 (60.19 %)
Age (years)	n=68
30-39	23 (33.82%)
40-49	26 (38.24%)
≥50	19 (27.94%)
Teaching time (years)	n=68
0-14	44 (64.71%)
15-29	21 (30.88%)
≥30	3 (4.41%)
Weekly work (h)	n=69
20	4 (5.8%)
40	65 (94.2%)
Work outside the office	n=68
Yes	33 (48.53%)
Sometimes	23 (33.82%)
No	12 (17.65%)
Work on the weekend	n=68
Yes	26 (38.34%)
Sometimes	29 (42.65%)
No	13 (19.12%)
Commute to work (min)	n=68
Up to 15	41 (60.29%)
Over 15	27 (39.71%)

**Figure 1:**
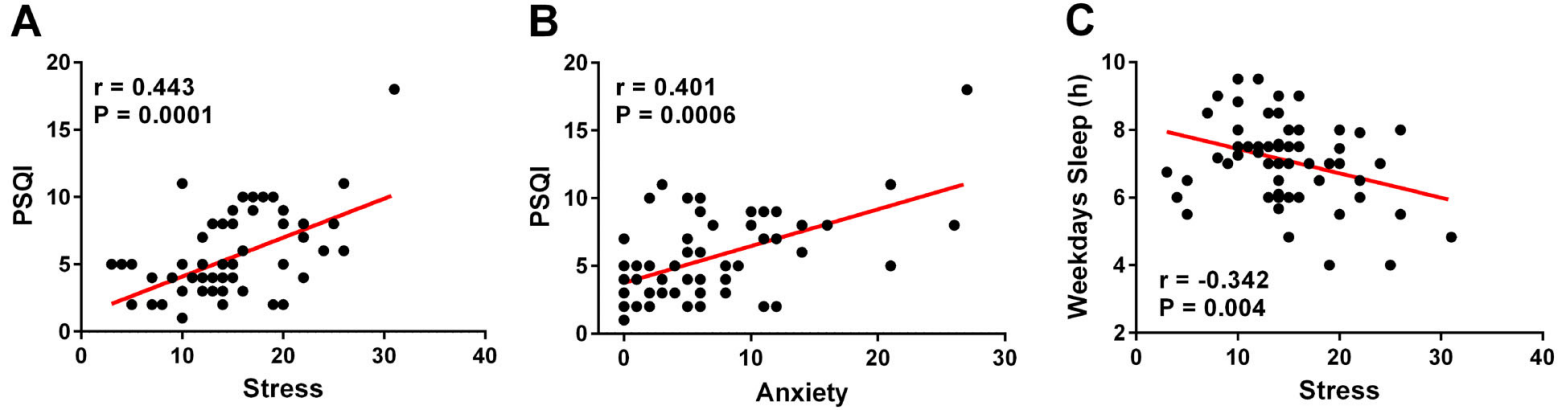
Relationship between stress and the Pittsburgh Sleep Quality Index (PSQI) (**A**), anxiety and PSQI (**B**), and stress and sleep duration on weekdays (**C**) in university professors. A and B, Spearman’s correlation; C, Pearson's correlation.

The mean scores for perceived stress scale and Beck's anxiety inventory were 14.8±11.7 and 6.3±0.7, respectively. Moreover, 26.4% of the professors self-reported psychological tension and stress. Other data related to the mental health of professors is shown in Table 2. We found an association between sleep quality and stress (r=0.44) and between sleep quality and anxiety (r=0.40) (Figure 1A and B). Similarly, stress (r=-0.34) negatively correlated with sleep duration on weekdays (Figure 1C).

**Table 2:** Mental health characteristics of university professors.

Characteristics	n (percentage)
Tension at work	n=67
None	3 (4.48%)
Very little	1 (1.49%)
Little	27 (40.3%)
Moderate	29 (43.28%)
Excessive	7 (10.45%)
Stress at work	n=67
Little	18 (26.87%)
Moderate	37 (55.22%)
Excessive	12 (17.91%)
Psychological follow-up	n=68
Yes	18 (26.47%)
No	50 (73.53%)
Perceived stress	n=68
Normal	13 (19.12%)
Low	51 (75%)
Moderate	3 (4.41%)
High	1 (1.47%)

The study revealed that 26% (n=45) of the professors presented a WC ≥88 cm and 12% of professors (n=12) had a WC ≥102 cm. The average HDL was 56.9±9.9 mg/dL (n=33), among which 30% of men (n=10) had HDL<40 mg/dL and 13% of women (n=23) had HDL <50 mg/dL. The mean level of triglycerides (n=41) was 115.5±18.0 mg/dL, whereas 19.5% of the subjects presented values higher than 150 mg/dL. The mean LDL cholesterol value was 123.5±26.9 (n=41) and 37.5% of the professors had values higher than 130 mg/dL. The general health characteristics of the participants is listed in Table 3. According to the National Cholesterol Education Program (NCEP) ATP III definition (20), metabolic syndrome is present if three or more of the following five criteria are met: WC over 40 inches (men) or 35 inches (women), blood pressure over 130/85 mmHg, fasting triglyceride (TG) level over 150 mg/dL, fasting HDL cholesterol level less than 40 mg/dL (men) or 50 mg/dL (women), and fasting blood sugar over 100 mg/dL.

**Table 3:** General health characteristics of participants.

Characteristics	n (percentage)	Mean (95%CI)
Smoker	n=68	
Yes	11 (16.18%)	
No	57 (83.82%)	
Comorbidities	n=68	
Yes	34 (50%)	
No	34 (50%)	
Alcohol consumption	n=67	
Yes	39 (58.2%)	
No	28 (41.8%)	
Systolic pressure (mmHg)	n=77	116.85 (113.42-120.29)
Glycemia (mg/dL)	n=48	91.85 (89.47-94.23)
HDL (mg/dL)	n=33	58.17 (34-93)
LDL (mg/dL)	n=32	123.56 (107.62-139.51)
Triglycerides (mg/dL)	n=41	115.51 (96.54-134.48)
BMI	n=79	25.68 (24.69-26.67)
Waist circumference (cm)	n=79	89.01 (86.21-91.81)

CI: confidence interval; HDL: high-density lipoprotein; LDL: low-density lipoprotein; BMI: body mass index.

Mean sleep duration (n=65) was 6.96±0.8 h. Most professors (83%) reported sleeping between 6 and 8 h, although 10.7% slept less than 6 h/night. The frequency of naps was 41.1% (n=69), being more frequent in men (22%) than in women (19.1%). The prevalence of poor sleep (41.2%; n=28) was higher in professors aged between 32 and 65 years, married (35.2%), and female (61.7%). Despite this finding, 85.2% of the participants were not under treatment with sleep medication at the time of data collection. The overall average score of PSQI was 5.48±0.6. Importantly, 23.5% of volunteers reported that they also worked overtime and 27.9% used to eat high calorie foods at night. We found no association of self-reported nocturnal eating with sleep parameters and cardiometabolic risk factors (arterial blood pressure, blood glucose concentration, HDL, LDL, triglycerides, BMI, WC; P>0.05). The general sleep parameters are shown in Table 4.

**Table 4:** General sleep parameters of university professors.

Characteristics	n (percent)	Mean (95%CI)
Sleep Quality	n=68	
Good	34 (50%)	
Poor	28 (41.18%)	
Sleep disorder	6 (8.82%)	
Social jetlag (min)	n=68	59.80 (50.81-68.80)
MSFsc (h)	n=68	2.86 (2.61-3.12)
Sleep on weekdays (h)	n=68	7.09 (6.80-7.37)
Sleep on weekends (h)	n=68	8.16 (7.85-8.47)
Sleep in previous month (h)	n=65	6.96 (6.69-7.22)
Latency (min)	n=66	19.51 (15.60-23.42)
Sleep efficiency	n=66	91.09 (86.45-95.73)
Waking up in the middle of the night or early in the morning	n=68	
Not once	14 (20.59%)	
Less than once a week	14 (20.59%)	
Once or twice a week	24 (35.29%)	
Three times a week or more	16 (23.53%)	
Taking sleeping medicine	n=68	
Not once	58 (85.29%)	
Less than once a week	5 (7.35%)	
Once or twice a week	2 (2.94%)	
Three times a week or more	3 (4.41%)	

MSFsc: Mid-sleep on free days corrected; CI: confidence interval.

**Figure 2:**
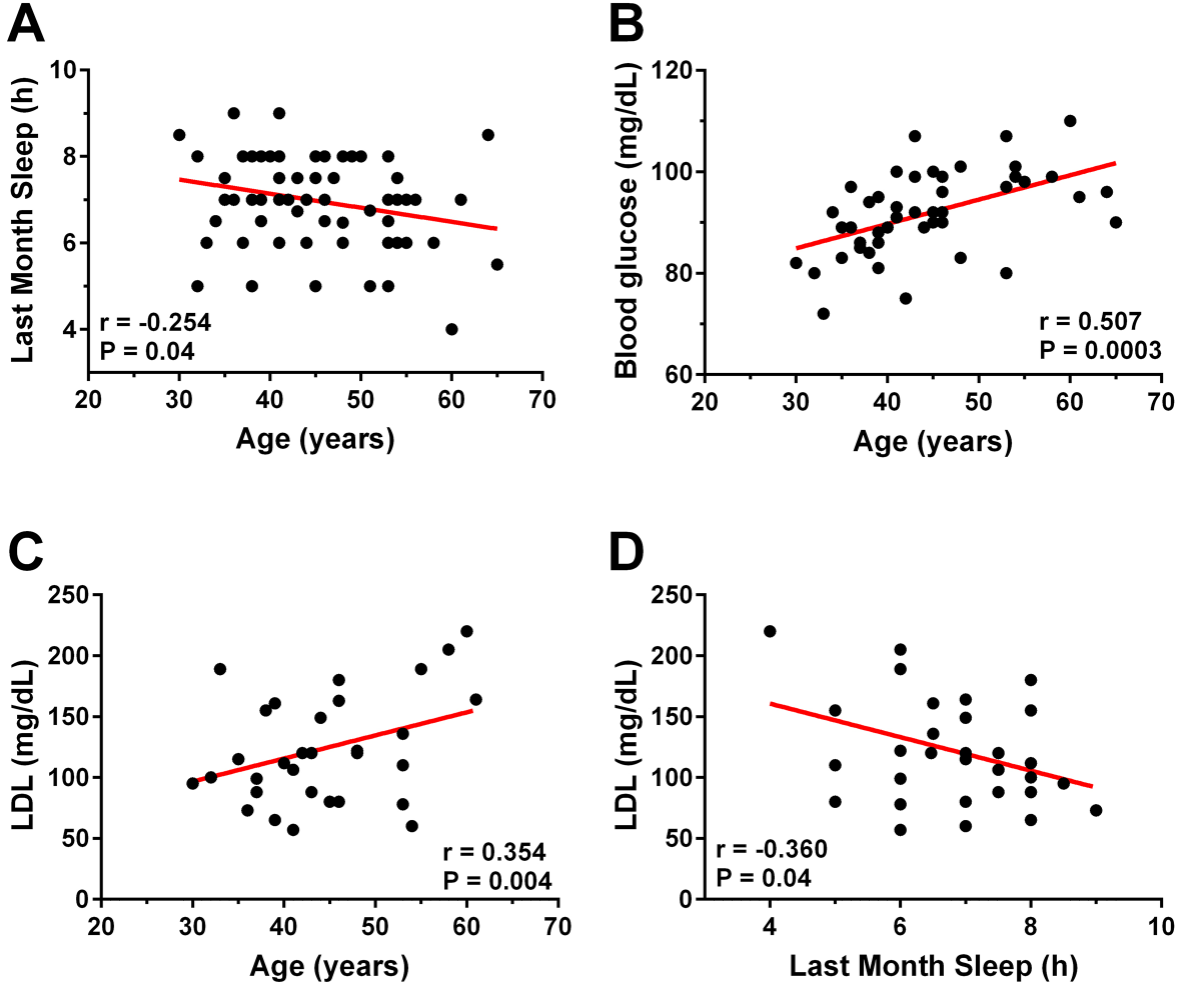
Correlations between age and sleep duration (**A**), age and blood glucose (**B**), age and low-density lipoprotein (LDL) cholesterol (**C**), and last month sleep and LDL cholesterol (**D**) (Pearson's correlation).

**Figure 3:**
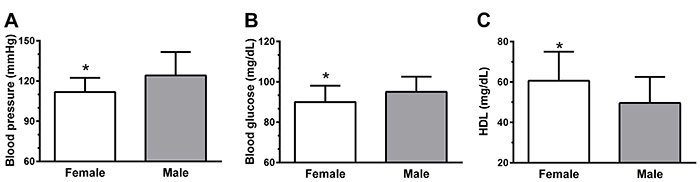
Cardiometabolic factors in female and male university professors. Systolic blood pressure (**A**), blood glucose (**B**), and high-density lipoprotein (HDL) cholesterol (**C**). Data are reported as means±SD. *P<0.05, Student's *t*-test for independent samples.

Professors who slept less in the previous month were significantly older (r=-0.25) (Figure 2A) and had high LDL cholesterol (r=-0.36) (Figure 2D). Age was also a determining factor for the prevalence of cardiometabolic risk in this population, due to the positive association between age and blood glucose concentration (r=0.51) and LDL cholesterol (r=0.35) (Figure 2B and C). Gender was also an important variable in this study, as it was observed that men presented higher systolic blood pressure (Figure 3A), blood glucose concentration (Figure 3B) and low serum HDL cholesterol (Figure 3C) compared to women (P<0.05).

**Figure 4:**
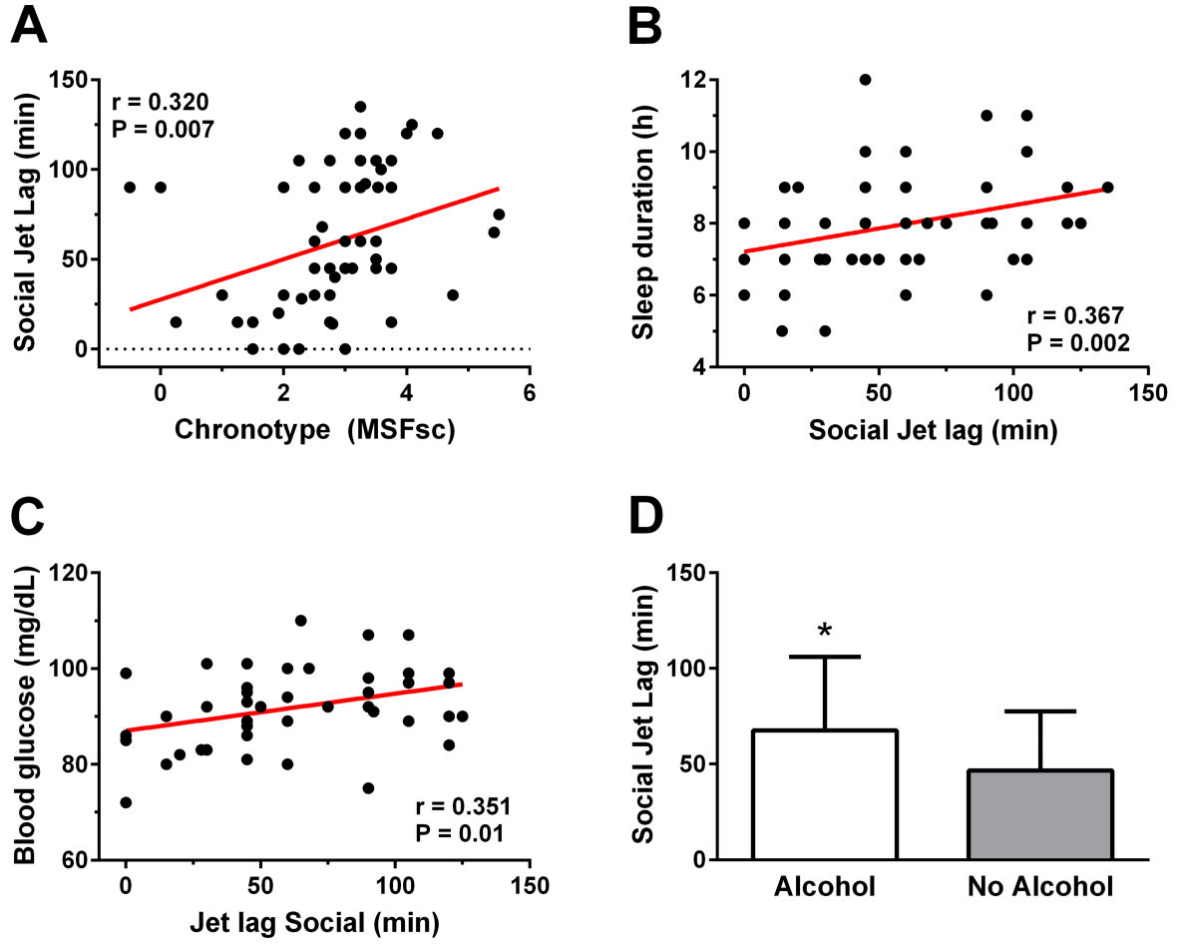
Relationship (Pearson’s correlation) between chronotype and social jet lag (**A**), social jet lag and weekend sleep (**B**), social jetlag and glycemia (**C**), and comparison of social jetlag between groups that drink alcohol or not (**D**). *P<0.05, Student's *t*-test. MSF: mid-sleep on free days.

In addition, this study showed an association between SJL and chronotype (r=0.32) (Figure 4A), SJL and weekend sleep (r=0.36) (Figure 4B), and SJL and blood glucose concentration (r=0.35) (Figure 4C). The correlations of SJL and other sleep parameters with cardiometabolic risk factors did not show significant results (P>0.05). Mean MSFsc was 183.7±22 min and mean SJL was 59.8±7.2 min (n=68), of which 51.4% had more than 1 h of SJL. Finally, SJL was higher in alcohol consumers (P<0.05) (Figure 4D).

## Discussion

We hypothesized that anxiety and stress in the work environment would be associated with SJL, poor sleep, and cardiometabolic risk factors among university professors. We found an association of sleep quality with stress and anxiety and of sleep duration with stress. In addition, SJL was positively associated with chronotype and cardiometabolic risk factors, for example, alcohol consumption and hyperglycemia. Sleep duration was also associated with lower LDL cholesterol.

These results corroborate previous findings that insomnia, poor sleep, short sleep duration, and excessive daytime sleepiness cause devastating effects on physical and psychological health (21). A recent study reported that 61% of high school teachers have poor sleep associated with high workload, depression, and stress (22). It was reported that the highest stress levels occurred in younger professors (aged between 36 and 45 years), with up to 15 years of teaching experience, with lower income and lower academic title, consistent with our findings, as we obtained comparable results from professors at initial and mid-career positions (1). According to the authors, these results can be explained by an increased tension related to competition for promotions among younger professors, who differ from older professors who are more familiar with academic work, which also contributes to the development of cardiometabolic diseases.

Studies in volunteers have shown that poor sleep is associated with markers of metabolic syndrome, such as hyperglycemia and low serum HDL cholesterol (23). An association between sleep duration and obesity was also highlighted, demonstrating that sleep deprivation leads to changes in appetite-regulating hormones, which explains these findings. The proportion of leptin and ghrelin levels, hormones that play a crucial role in the central control of appetite and energy expenditure, has been reported as a pathophysiological mechanism that links short sleep duration to obesity (24). Sleep is an important modulator of hormonal secretion, glucose regulation, and cardiovascular function, and there is a negative relationship between sleep duration and degree of metabolic syndrome (25,26).

The current study reaffirmed the relationship between age and metabolic alteration. Equivalent results were seen in a meta-analysis involving professors, which found higher cardiometabolic risk factors among older men, which were inversely associated with sleep duration (25). Under physiological conditions, men tend to suffer cardiovascular comorbidities earlier than women (27).

The results of this study also demonstrated that occupational demands on weekdays make it impossible for professors to respect their biological sleep preferences, with a misalignment between chronotype and social demands that impacts sleep duration and leads to this sleep debt being paid on weekends or days off. In this sense, the risk of occurrence of SJL may be higher depending on work activity, such as workload and work timing. Studies have shown that sleep improves when working hours agree with chronotype (28). The presence of SJL contributes to the causal link between sleep deprivation, shift work, and cardiometabolic alterations (7,29).

The literature has also highlighted that SJL is associated with worse mental and physical health, resulting in poor academic performance (30). One of the biological determinants of the emergence of SJL is chronotype. Individuals with an evening chronotype prefer doing activities at a later time of the day (31).

The correlation between chronotype and SJL in the present study was not surprising. On weekends, evening chronotypes sleep later and extend sleep duration (7). It is now a consensus that there is an association between greater SJL and school times in adolescents with evening chronotype (32). This association does not seem to be restricted to students, occurring also with professors, leading to short sleep duration, higher SJL, and poor sleep quality.

As observed in our results, SJL was positively correlated with alcohol consumption. Previous research considered alcohol consumption as a “nocturnal behavior”, and found that evening chronotypes, which have more SJL than morning ones, have more opportunities to experience this risk behavior (33). These authors also considered that individuals who have higher SJL use these substances more frequently to deal with stress triggered by the misalignment between the biological clock and the social clock. Alcohol use, for example, may reduce sleep latency but subsequently disrupt sleep architecture by increasing the amount of non-rapid eye movement (NREM) sleep during the first half of the night and interrupting the rapid eye movement (REM) phase in the second half (34).

Evening types are more likely to have inadequate eating habits, such as nocturnal caloric meals and fast-food plus soft drink consumption, therefore ending up with higher BMI and glycemic values (34). Thus, changes in the circadian system, such as those related to SJL, influence these unhealthy habits, and are associated with higher incidence of obesity and metabolic disorders (30).

Our results were consistent with previous studies, showing that sleep irregularities (weekdays *vs* weekends) influence the physiological pathways that regulate energy metabolism (35). Considering sleep as an adjustable risk factor, the results suggested that strategies can be implemented to improve the amount and quality of sleep of university professors and prevent the development of diseases. Moreover, this research also intended to sensitize institutions to organize professors’ activities according to their chronotype, therefore mitigating stress, anxiety, and possible sleep disorders.

One of the major limitations of this study was the discrepancy of the number of subjects for each variable. Most volunteers completed the online questionnaires but did not attend the physical assessment session and did not deliver the complementary exams. Further studies are necessary to confirm these findings, including longitudinal studies involving university professors.

In conclusion, despite the small sample, this was the first study to confirm the hypothesis that stress and anxiety in university professors of Rio Grande do Norte are negatively associated with sleep quality and sleep duration as well as with cardiometabolic risks related to SJL. This study might be a cost- and health-effective approach to predict cardiovascular diseases in university professors. Actions and strategies to improve sleep and promote health benefits are necessary to guarantee quality of life, health, and performance of university professors.

## References

[1] Li W, Kou C (2018). Prevalence and correlates of psychological stress among teachers at a national key comprehensive university in China. Int J Occup Environ Health.

[2] Chellappa SL, Vujovic N, Williams JS, Scheer FAJL (2019). Impact of circadian disruption on cardiovascular function and disease. Trends Endocrinol Metab.

[3] Zimmet P, Alberti KGMM, Stern N, Bilu C, El-Osta A, Einat H (2019). The circadian syndrome: is the metabolic syndrome and much more!. J Intern Med.

[4] Smith SM, Vale WW (2006). The role of the hypothalamic-pituitary-adrenal axis in neuroendocrine responses to stress. Dialogues Clin Neurosci.

[5] Collie RJ, Shapka JD, Perry NE (2012). School climate and social-emotional learning: predicting professor stress, job satisfaction, and teaching efficacy. J Educ Psychol.

[6] Reutrakul S, Van Cauter E (2014). Interactions between sleep. circadian function. and glucose metabolism: implications for risk and severity of diabetes. Ann NY Acad Sci.

[7] Roenneberg T, Pilz LK, Zerbini G, Winnebeck EC (2019). Chronotype and social jetlag: a (self-) critical review. Biology (Basel).

[8] Watson NF, Badr MS, Belenky G, Bliwise DL, Buxton OM, Buysse D (2015). Recommended amount of sleep for a healthy adult: a joint consensus statement of the American Academy of Sleep Medicine and Sleep Research Society. Sleep.

[9] Kivimäki M, Steptoe A (2018). Effects of stress on the development and progression of cardiovascular disease. Nat Rev Cardiol.

[10] Bertisch SM, Pollock BD, Mittleman MA, Buysse DJ, Bazzano LA, Gottlieb DJ (2018). Insomnia with objective short sleep duration and risk of incident cardiovascular disease and all-cause mortality: sleep heart health study. Sleep.

[11] Pigeon WR, Bishop TM, Krueger KM (2017). Insomnia as a precipitating factor in new onset mental illness: a systematic review of recent findings. Curr Psychiatry Rep.

[12] Kalmbach DA, Anderson JR, Drake CL (2018). The impact of stress on sleep: Pathogenic sleep reactivity as a vulnerability to insomnia and circadian disorders. J Sleep Res.

[13] Huang T, Redline S (2019). Cross-sectional and prospective associations of actigraphy-assessed sleep regularity with metabolic abnormalities: the multi-ethnic study of atherosclerosis. Diabetes Care.

[14] Parsons MJ, Moffitt TE, Gregory AM, Goldman-Mellor S, Nolan PM, Poulton R (2015). Social jetlag, obesity and metabolic disorder: investigation in a cohort study. Int J Obes (Lond).

[15] Lo JC, Leong RLF, Loh KK, Dijk DJ (2014). Chee MWL. Young adults' sleep duration on work days:differences between east and west. Front Neurol.

[16] Poggiogalle E, Jamshed H, Peterson CM (2018). Circadian regulation of glucose, Lipid, and energy metabolism in humans. Metabolism.

[17] Bertolazi AN, Fagondes SC, Hoff LS, Dartora EG, Miozzo CS, Barba MEF (2011). Validation of the Brazilian Portuguese version of the Pittsburgh sleep quality index. Sleep Med.

[18] Cohen S, Kamarck T, Mermelstein R (1983). A global measure of perceived stress. J Health Soc Behav.

[19] Cunha JA (2001). Manual da versão em português das Escalas Beck.

[20] Expert Panel on Detection, Evaluation, and Treatment of High Blood Cholesterol in Adults (2001). Executive summary of the third report of the National Cholesterol Education Program (NCEP) expert panel on detection, evaluation, and treatment of high blood cholesterol in adults (adult treatment panel iii). JAMA.

[21] Lao XQ, Liu X, Deng HB, Chan TC, Ho KF, Wang F (2018). Sleep quality, sleep duration, and the risk of coronary heart disease: a prospective cohort study with 60,586 adults. J Clin Sleep Med.

[22] Musa NA, Moy FM, Wong LP (2018). Prevalence and factors associated with poor sleep quality among secondary school teachers in a developing country. Ind Health.

[23] Hung HC, Yang YC, Ou HY, Wu JS, Lu FH, Chang CJ (2013). The association between self-reported sleep quality and metabolic syndrome. PLoS One.

[24] Taheri S, Lin L, Austin D, Young T, Mignot E (2004). Short sleep duration is associated with reduced leptin, elevated ghrelin, and increased body mass index. PLoS Med.

[25] Iftikhar IH, Donley MA, Mindel J, Pleister A, Soriano S, Magalang UJ (2015). Sleep duration and metabolic syndrome, an updated dose-risk metaanalysis. Ann Am Thorac Soc.

[26] Syauqy A, Hsu CY, Rau HH, Kurniawan AL, Chao JCJ (2019). Association of sleep duration and insomnia symptoms with components of metabolic syndrome and inflammation in middle-aged and older adults with metabolic syndrome in Taiwan. Nutrients.

[27] Kander MC, Cui Y, Liu Z (2017). Gender difference in oxidative stress: a new look at the mechanisms for cardiovascular diseases. J Cell Mol Med.

[28] Vetter C, Fischer D, Matera JL, Roenneberg T (2015). Aligning work and circadian time in shift workers improves sleep and reduces circadian disruption. Curr Biol.

[29] Wong PM, Hasler BP, Kamarck TW, Muldoon MF, Manuck SB (2015). Social jetlag chronotype and cardiometabolic risk. J Clin Endocrinol Metab.

[30] Beauvalet JC, Quiles C, De Oliveira MAB, Ilgenfritz CAV, Hidalgo MPL, Tonon AC (2017). Social jetlag in health and behavioral research: a systematic review. ChronoPhysiol Ther.

[31] Anothaisintawee T, Lertrattananon D, Thamakaison T, Thakkinstian A, Reutrakul S (2018). The relationship among morningness-eveningness, sleep duration, social jetlag, and body mass index in Asian patients with prediabetes. Front Endocrinol (Lausanne).

[32] Randler C, Vollmer C, Kalb N, Itzek-Greulich H (2019). Breakpoints of time in bed. midpoint of sleep. and social jetlag from infancy to early adulthood. Sleep Med.

[33] Wittmann M, Dinich J, Merrow M, Roenneberg T (2006). Social jetlag: misalignment of biological and social time. Chronobiol Int.

[34] Chakravorty S, Chaudhary NS, Brower KJ (2016). Alcohol dependence and its relationship with insomnia and other sleep disorders, alcohol. Clin Exp Res.

[35] Cipolla-Neto J, Amaral FG, Afeche SC, Tan DX, Reiter RJ (2014). Melatonin, energy metabolism, and obesity: a review. J Pineal Res.

